# Tuberculous osteomyelitis of the midfoot: a case report

**DOI:** 10.4076/1757-1626-2-6859

**Published:** 2009-07-07

**Authors:** Julia D Flint, Shanmugam Saravana

**Affiliations:** Department of Rheumatology, City HospitalBirminghamUK

## Abstract

**Introduction:**

The prevalence of tuberculosis is increasing and musculoskeletal tuberculosis, although currently rare, may become an important problem.

**Case presentation:**

We report the case of a 20-year-old Somalian man, who presented with an inversion injury to his ankle. When further history was taken, it transpired that he had sustained trauma to his ankle in Somalia 4 years previously, complicated by a non-healing wound. His foot pain and swelling had been present ever since. Diagnosis of tuberculosis was made by bone biopsy, histology of which demonstrated caseating granulomas. Tissue culture yielded growth of tuberculous bacilli. The patient made a full recovery on anti-tuberculous treatment.

**Conclusion:**

Musculoskeletal tuberculosis can be difficult to diagnose as only about one third of patients have respiratory symptoms. Synovial fluid aspirate is relatively unlikely to lead to definitive diagnosis, and a bone biopsy should always be taken for culture and histological examination.

## Introduction

Tuberculosis is a major global health problem, with an estimated incidence of 9.2 million cases worldwide in 2006 [1]. Prevalence of TB in the UK has increased from 9/100000 in 1990 to 12/100000 in 2001 [1]. Musculoskeletal tuberculosis accounts for 1-2% of all cases of tuberculosis in the Western World [2]. The spine is most commonly affected, and foot involvement is rare [3].

## Case presentation

We report the case of a 20-year-old Somalian man, who presented with an inversion injury of his ankle, sustained during a fall downstairs. He had subsequent pain in the foot and difficulty walking. The history on admission was limited due to language difficulties, but he felt otherwise generally well.

**Figure 1.**
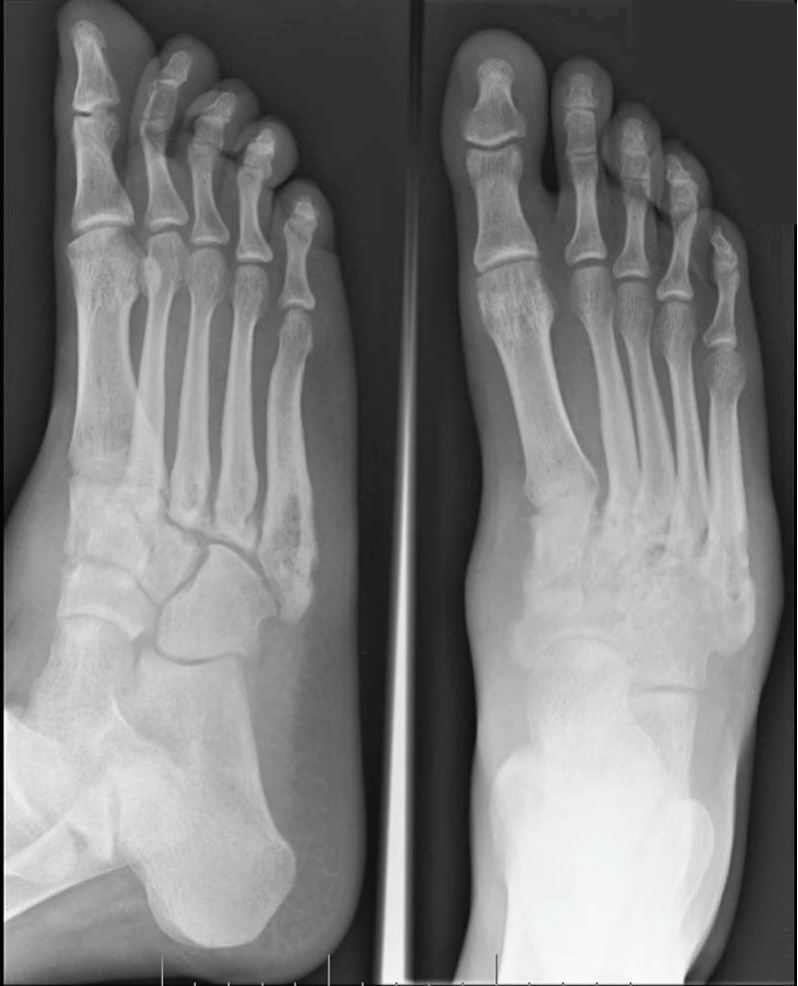
Right foot x-ray on day of admission - 2 views. Abnormal lucent lesions are seen at the base of the 3^rd^, 4^th^ and 5^th^ metatarsals.

Examination demonstrated tenderness over the base of the 1^st^ and 5^th^ metatarsals, cuboid and navicular, with swelling of the dorsal lateral aspect of the right foot. Ankle movements were normal, although limited by pain. There was warmth of the overlying skin, but no rash was seen. No abnormalities were detected in other joints, and systemic examination was normal with no lymphadenopathy or fever and unremarkable respiratory, cardiovascular and abdominal examinations. He had a slightly raised CRP of 10, normal ESR of 10, and an x-ray of the right foot and ankle showed abnormal lucent lesions at the base of the 3^rd^, 4^th^ and 5^th^ metatarsals but no recent fracture (Figure 1).

Further history was finally obtained through an interpreter. The patient had come to the UK 2 years previously, but had sustained trauma to his foot during a road traffic accident in Somalia about 4 years previously. Following this he had persistent foot pain and a non-healing wound. He had not sought medical attention until 2 years later when he had presented to an orthopaedic outpatient clinic. An x-ray performed on this occasion demonstrated deformities at the bases of the lateral four metatarsal bones and of the lateral cuneiform bone. These deformities were well corticated and attributed to old fractures. He reported no contacts with TB and on direct questioning had no respiratory symptoms, fevers, night sweats or weight loss, and no other past medical history. Although bloods revealed a concomitant hepatitis B infection, an HIV test was negative.

**Figure 2.**
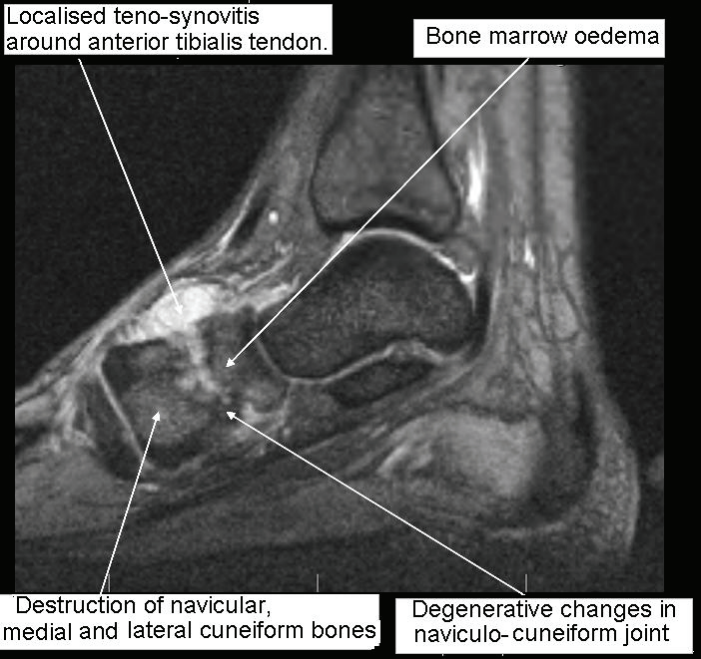
MRI scan right foot, T2 weighted, 5 days after admission. Marked degenerative changes are seen in the naviculo-cuneiform joint. There is an associated joint effusion extending to dorsal aspect of mid foot causing a prominent localised teno-synovitis around the anterior tibialis tendon.

An MRI scan showed prominent bone marrow oedema with destruction of the navicular, medial and lateral and cuneiform bones (Figure 2). Marked degenerative changes were seen in the naviculo-cuneiform joint with associated joint effusion extending to dorsal aspect of mid foot causing a prominent localised teno-synovitis around the anterior tibialis tendon. The overall appearances were suggestive of either osteomyelitis with associated septic arthritis, bony tumour or neuropathic joint. There was insufficient fluid to aspirate under ultrasound guidance, and the patient underwent a bone biopsy. Histology of the bone core demonstrated multiple granulomas, some of which showed ‘a hint of caseation’. Although ZN and fungal stains were negative, subsequent tissue culture grew mycobacterium tuberculosis.

The patient was started on quadruple anti-tuberculous therapy for 2 months: rifampicin 720 mg, isoniazid 300 mg, pyrizinamide 1800 mg (in combination as ‘Rifater’), and ethambutol 300 mg. He was given prophylactic vitamin B6 (pyridoxine 10 mg), and after 2 months ethambutol and pyrizinamide were discontinued in line with the standard treatment regime. Within 4 months of starting treatment the patient was pain free, fully weight bearing on the affected foot, and x-rays showed no further bony destruction.

## Discussion

Musculoskeletal tuberculosis is difficult to diagnose. The classic presentation with localised pain, together with fever and weight loss is rarely seen. Blood tests may show a raised ESR [4]. Only about one third of patients with musculoskeletal tuberculosis have pulmonary involvement [5], making chest x-ray screening less useful.

Radiological features of musculoskeletal tuberculosis are non specific, but may include bone marrow oedema, osteoporosis or lytic lesions. The surrounding tissue may show synovitis, joint effusions, tenosynovitis, soft tissue collections, or myositis [6,7]. MRI appearances are non-specific and may be consistent with osteomyelitis, bony tumour, avascular necrosis or a neuropathic joint, but if tuberculosis is a possibility, a tissue diagnosis should be sought. Synovial fluid aspirate is relatively unlikely to lead to definitive diagnosis, and a bone biopsy should also be taken for microscopy, culture and histology [3]. Tuberculous bacilli are rarely seen (with Ziehl-Neelsen staining) or grown in culture, and the diagnosis often has to be made based on the granulomatous appearance histologically along with high clinical suspicion.

Drug therapy is usually sufficient to treat tubercular osteomyelitis, although surgery is very occasionally required [7]. Isolated osteomyelitis can be seen in the early stages of the disease, but early treatment is required to prevent possible sequelae, such as septic arthritis of adjacent joints [8]. Quadruple anti-tuberculous therapy should be commenced as soon as the diagnosis is made. The usual regime is 2 months daily treatment with rifampicin, isoniazid, pyrazinamide and ethambutol. Rifampicin and isoniazid are continued for a further 4 months. Notification of every new diagnosis of tuberculosis must be sent to the health protection agency.

This patient was diagnosed with tuberculous osteomyelitis of the mid foot. The history of inversion injury was in fact a red-herring in this case as the tuberculosis had presumably been contracted following the trauma and open wound sustained 4 years previously. The importance of getting a full history, through an interpreter where necessary is clearly demonstrated by this case.

## Conclusion

Tuberculosis is on the rise and it is important to recognise the less common presentations of this condition to enable early diagnosis and successful treatment. In inner city areas with a high prevalence of TB, there may be no history of TB contact, or any ‘typical’ symptoms or signs. Radiological features are non-specific and so a high index of suspicion is required. In patients with risk factors, or any suspicion of TB, a biopsy should be performed to make the diagnosis and initiate treatment.

## Abbreviations

MRI: Magnetic resonance imaging
TB: Tuberculosis
ZN: Zeil Neelson stain
